# Travel time to emergency care not by geographic time, but by optimal time: A nationwide cross-sectional study for establishing optimal hospital access time to emergency medical care in South Korea

**DOI:** 10.1371/journal.pone.0251116

**Published:** 2021-05-03

**Authors:** Won Mo Jang, Juyeon Lee, Sang Jun Eun, Jun Yim, Yoon Kim, Mi Young Kwak

**Affiliations:** 1 Department of Public Health and Community Medicine, Seoul Metropolitan Government-Seoul National University Boramae Medical Center, Seoul, South Korea; 2 Center for Public Health, National Medical Center, Seoul, South Korea; 3 Department of Preventive Medicine, Chungnam National University College of Medicine, Daejeon, South Korea; 4 Department of Citizen Health, University of Seoul, Seoul, South Korea; 5 Institute of Health Policy and Management, Medical Research Center, Seoul National University, Seoul, South Korea; 6 Department of Health Policy and Management, Seoul National University College of Medicine, Seoul, South Korea; University of Georgia, UNITED STATES

## Abstract

Increase in travel time, beyond a critical point, to emergency care may lead to a residential disparity in the outcome of patients with acute conditions. However, few studies have evaluated the evidence of travel time benchmarks in view of the association between travel time and outcome. Thus, this study aimed to establish the optimal hospital access time (OHAT) for emergency care in South Korea. We used nationwide healthcare claims data collected by the National Health Insurance System database of South Korea. Claims data of 445,548 patients who had visited emergency centers between January 1, 2006 and December 31, 2014 were analyzed. Travel time, by vehicle from the residence of the patient, to the emergency center was calculated. Thirteen emergency care–sensitive conditions (ECSCs) were selected by a multidisciplinary expert panel. The 30-day mortality after discharge was set as the outcome measure of emergency care. A change-point analysis was performed to identify the threshold where the mortality of ECSCs changed significantly. The differences in risk-adjusted mortality between patients living outside of OHAT and those living inside OHAT were evaluated. Five ECSCs showed a significant threshold where the mortality changed according to their OHAT. These were intracranial injury, acute myocardial infarction, other acute ischemic heart disease, fracture of the femur, and sepsis. The calculated OHAT were 71–80 min, 31–40 min, 70–80 min, 41–50 min, and 61–70 min, respectively. Those who lived outside the OHAT had higher risks of death, even after adjustment (adjusted OR: 1.04–7.21; 95% CI: 1.03–26.34). In conclusion, the OHAT for emergency care with no significant increase in mortality is in the 31–80 min range. Optimal travel time to hospital should be established by optimal time for outcomes, and not by geographic time, to resolve the disparities in geographical accessibility to emergency care.

## Introduction

Emergency care can be the last opportunity to prevent mortality in both patients with acute conditions and worsened chronic diseases. In this aspect, emergency care is considered a basic human right as stipulated in the General Comment No. 14: The Right to the Highest Attainable Standard of the Office of the United Nations High Commissioner for Human Rights [1]. If accessibility is accepted as a core element of right to emergency care, equitable distribution of emergency care facilities is an important strategy to implement the right to emergency care.

Distance to emergency care without any intervention can also disproportionately affect timely access to care and may lead to residential disparities in the outcome of care in developed countries. The distance decay effect, that is, a lower usage rate of emergency facilities among those who live farther, after adjustment for need, than those who live closer, has been reported in developed countries [2–6]. With regard to the outcomes of emergency care, a negative association was also observed between the outcomes of services and distance within the context of developed countries [7]. Increased travel time to emergency care facilities is associated with a higher mortality rate among patients with acute cardiovascular conditions [8–10] and worse outcomes in patients with severe injuries [11, 12].

Given the marked influence of distance on the outcomes of emergency care, many countries have attempted to solve the problem with respect to the geospatial approach by planning the allocation of facilities. Since 2017, the South Korean government has made efforts to enhance geographic accessibility to emergency care facilities by vulnerable area selection, rather than the population size-oriented approach, in accordance with the Public Health and Medical Services Act [13]. Vulnerable areas were defined as areas where access to local emergency medical centers is within 30 min or regional emergency medical centers is within 60 min from residence.

South Korea has 17 high-level local administrative territories (one Seoul metropolitan city, six metropolitan cities, one self-governing city, and nine provinces) and 260 low-level local administrative districts (Si, Gun, and Gu) nationwide. The Korean Emergency Medical Service Act identified 38 regional emergency medical centers, 124 local emergency medical centers, 240 local emergency medical institutes, 20 special emergency medical centers, and 119 non-designated emergency medical facilities in 2019 [14]. More than 38% of low-level local administrative districts (99 Si, Gun, and Gu) were classified as vulnerable areas of emergency medical care in the same year [15].

However, few studies have attempted to evaluate the evidence of travel time benchmarks for selection of emergency care vulnerable areas in view of the association between travel time and outcomes [8–12, 15, 16]. Identifying the optimal access time to emergency medical care based on the travel time-outcome relationship would help to lower the adverse emergency care outcomes that result from environmental obstacles. Thus, this study aimed to establish the optimal hospital access time (OHAT) for emergency care in South Korea.

## Materials and methods

### Study design and population

This was a retrospective cross-sectional study using National Health Insurance System (NHIS) claims database of South Korea. We included all hospitalized patients with emergency care–sensitive conditions (ECSC) who had been admitted via the emergency room of the regional emergency medical centers or tertiary university hospitals center between January 1, 2006 and December 31, 2014. Patients who received palliative therapy and with missing or unclear information on age, address, and date of death were excluded.

The NHIS was established in 1989 in South Korea to achieve the goal of universal healthcare. The NHIS is also involved in reviewing the Medical Aid beneficiaries. Accordingly, the NHIS claims database comprises of claims data, including emergency claims, from both the National Health Insurance and Medical Aid submitted by health care providers. It is nationally representative of the population’s medical utilization. The NHIS claims database includes information about residence of the patient, age, sex, income-based insurance contributions, health care utilization (length of stay, diagnoses, procedures, operations, pharmaceuticals, cost, location of hospitals), and date of death. However, means of transport, conditions of traffic to emergency medical facilities, and time of event are lacking in the NHIS claims database.

### Selection of emergency care–sensitive conditions and outcome variable

To identify the ECSCs, 40 candidate conditions were collected based on previous studies on ECSC [16–18]. Then, a multidisciplinary expert panel involving academic emergency physicians, nurses, and health service researchers reviewed the lists of ECSC. Finally 13 ECSCs projected to have a higher time sensitivities than the others were selected through group discussions. Table 1 shows the detailed definition based on the International Classification of Diseases-10 (ICD-10). The outcome was defined as death occurring within 30 days after discharge, but death on hospital arrival was not excluded in the analysis due to the data limitations. The study population comprised of 445,548 patients; of them, 105,739 and 339,809 did and did not have injuries, respectively.

**Table 1:** ICD codes of the 13 emergency care–sensitive conditions.

ECSC	ICD-10 code
Cardiac arrest	I46 (I460-I469)
Intracranial injury	S06 (S060-S069)
Subarachnoid hemorrhage	I60 (I600-I609)
Intracerebral hemorrhage	I61 (I610-I619)
Other nontraumatic intracranial hemorrhage	I62 (I620-I629)
Cerebral infarction	I63 (I630-I639)
Stroke, not specified as hemorrhage or infarction	I64 (I640-I649)
Heart failure	I50 (I500-I509)
Acute myocardial infarction	I21 (I210-I219)
Other acute ischemic heart disease	I24 (I240-I249)
Peritonitis	K65 (K650-K659)
Fracture of the femur	S72 (S720-S729)
Sepsis	A41 (A410-A419)

ICD, International Classification of Diseases; ECSC, emergency care–sensitive condition

### Calculation of travel time to hospital

We obtained the location of the emergency care facilities and the patient address from the NHIS database. In this study, travel time to the hospital was defined as the driving time by motor vehicle from the patient’s home to the emergency care facility. The Ministry of Land, Infrastructure and Transport (MOLIT) network analysis system was used to investigate the travel time to the hospital based on a geographic information system (GIS) package that includes a national driving time matrix. The National Spatial Data Infrastructure Portal (http://www.nsdi.go.kr/lxmap/index.do) was developed by MOLIT for the national land space planning by integrating land information work with GIS. The travel time to the hospital was computed using the location information from NHIS and GIS developed by MOLIT.

### Assessment of optimal hospital access time

The OHAT was defined as the first significant increase in risk-adjusted mortality of the ECSCs. The change-point analysis (CPA) tool developed by Taylor was performed to determine the OHAT. The CPA is a useful tool for identifying non-periodic events and determine whether a change has occurred in a time series dataset by providing the confidence level and confidence interval [19–21]. In brief, CPA requires iterative procedures consisting of a combination of cumulative sum charts (CUSUM) and bootstrapping to identify the significant changes. Bootstrapping was performed as follows. First, we generated a bootstrap sample of data at 10-min units. Then, based on the bootstrap sample, the bootstrap CUSUM, denoted as 10 min, 20…, 350 min, was calculated. Next, maximum, minimum, and difference of the bootstrap CUSUM was calculated. To detect a significant change point, the confidence level should be calculated. For example, if 995 of the 1,000 bootstraps had a significant change, the confidence level is $\left(Confidence\;level=100*\frac{995}{1000}\%=95\%\right)$.

### Statistical analysis

We adopted the OHAT of each ECSC and created another location dummy variable to distinguish between patients inside and outside the OHAT. The location dummy variable was added to the risk adjustment mortality model used in the previous steps for OHAT analysis. The differences in the risk-adjusted mortality rate between patients living inside and outside the OHAT were determined using the odds ratio. To determine the differences in risk-adjusted mortality between patients who live within and outside the OHAT, the risk-adjusted mortality rate of each ECSC was analyzed using multiple logistic regression adjusted for age, socioeconomic status, Charlson Comorbidity Index or Excess Mortality Ratio-adjusted Injury Severity Score, urbanization, and size of the hospital. To apply the appropriate risk adjustment according to the type of acute condition, we used the Charlson Comorbidity Index for non-traumatic diseases and the Excess Mortality Ratio-adjusted Injury Severity Score for traumatic diseases (e.g., intracranial injury, fracture of the femur) [22–26]. The risk adjusted mortality rate (deaths per 1,000 patients) was plotted against the travel time to hospital at 10-min intervals to determine the OHAT for each ECSC. All statistical analyses were conducted using SAS, version 10.2 (SAS Institute Inc, Cary, NC). All P values were two tailed, and P <0.05 was considered significant.

### Ethics

The study was reviewed and approved by the Institutional Review Board of Seoul National University College of Medicine (IRB No. E-1504-075-665). The requirement for informed consent was waived.

## Results

In total, 378,324 patients (84.9%) survived, while 67,224 patients (15.1%) died within 30 days after discharge (Table 2). Compared with non-survivors, the survivors were more likely to live in the metropolitan area and be covered by NHI. Of the 13 ECSCs, sepsis showed the highest rate of mortality.

**Table 2:** Patient characteristics.

Variables		Survivors	Non-survivors	Total
Total		378,324 (84.9)	67,224 (15.1)	445,548 (100.0)
Sex*				
	Male	220,810 (86.0)	35,880 (14.0)	256,690 (100.0)
	Female	157,514 (83.4)	31,344 (16.6)	188,858 (100.0)
Age, years*				
	0–9	22,721 (98.4%)	361 (1.6%)	23,082 (100.0)
	10–29	16,071 (94.6%)	910 (5.4%)	16,981 (100.0)
	30–49	63,698 (90.6%)	6,579 (9.4%)	70,277 (100.0)
	50–69	155,523 (88.4%)	20,319 (11.6%)	175,842 (100.0)
	≥70	120,311 (75.5%)	39,055 (24.5%)	159,366 (100.0)
Area of residence*				
	Metropolitan	216,342 (85.8)	35,947 (14.2)	252,289 (100.0)
	City	108,177 (84.1)	20,457 (15.9)	128,634 (100.0)
	Rural	53,805 (83.3)	10,820 (16.7)	64,625 (100.0)
Type of insurance*				
	NHI	353,121 (85.7)	58,836 (14.3)	411,957 (100.0)
	MA	25,203 (75.0)	8,388 (25.0)	33,591 (100.0)
ECSC*				
	Cardiac arrest	1,932 (30.0)	45,16 (70.0)	6,448 (100.0)
	Intracranial injury	60,743 (90.4)	6,467 (9.6)	67,210 (100.0)
	Subarachnoid hemorrhage	17,457 (72.8)	6,512 (27.2)	23,969 (100.0)
	Intracerebral hemorrhage	21,128 (71.9)	8,254 (28.1)	29,382 (100.0)
	Other nontraumatic intracranial hemorrhage	5,668 (80.8)	1,343 (19.2)	7,011 (100.0)
	Cerebral infarction	118,637 (91.5)	10,956 (8.5)	129,593 (100.0)
	Stroke, not specified as hemorrhage or infarction	3,353 (92.8)	260 (7.2)	3,613 (100.0)
	Heart failure	14,056 (78.5)	3,850 (21.5)	17,906 (100.0)
	Acute myocardial infarction	64,539 (89.4)	7,656 (10.6)	72,195 (100.0)
	Other acute ischemic heart disease	820 (86.0)	133 (14.0)	953 (100.0)
	Peritonitis	9,082 (85.1)	1,586 (14.9)	10,668 (100.0)
	Fracture of the femur	36,570 (94.9)	1,959 (5.1)	38,529 (100.0)
	Sepsis	24,339 (63.9)	13,732 (36.1)	38,071 (100.0)

The data are presented in n (%) format.

NHI: National Health Insurance, MA: Medical Aid, SD: standard deviation, ECSC: emergency care–sensitive condition

*p<0.05 calculated by chi-square test between survival and death groups

**Fig 1:**
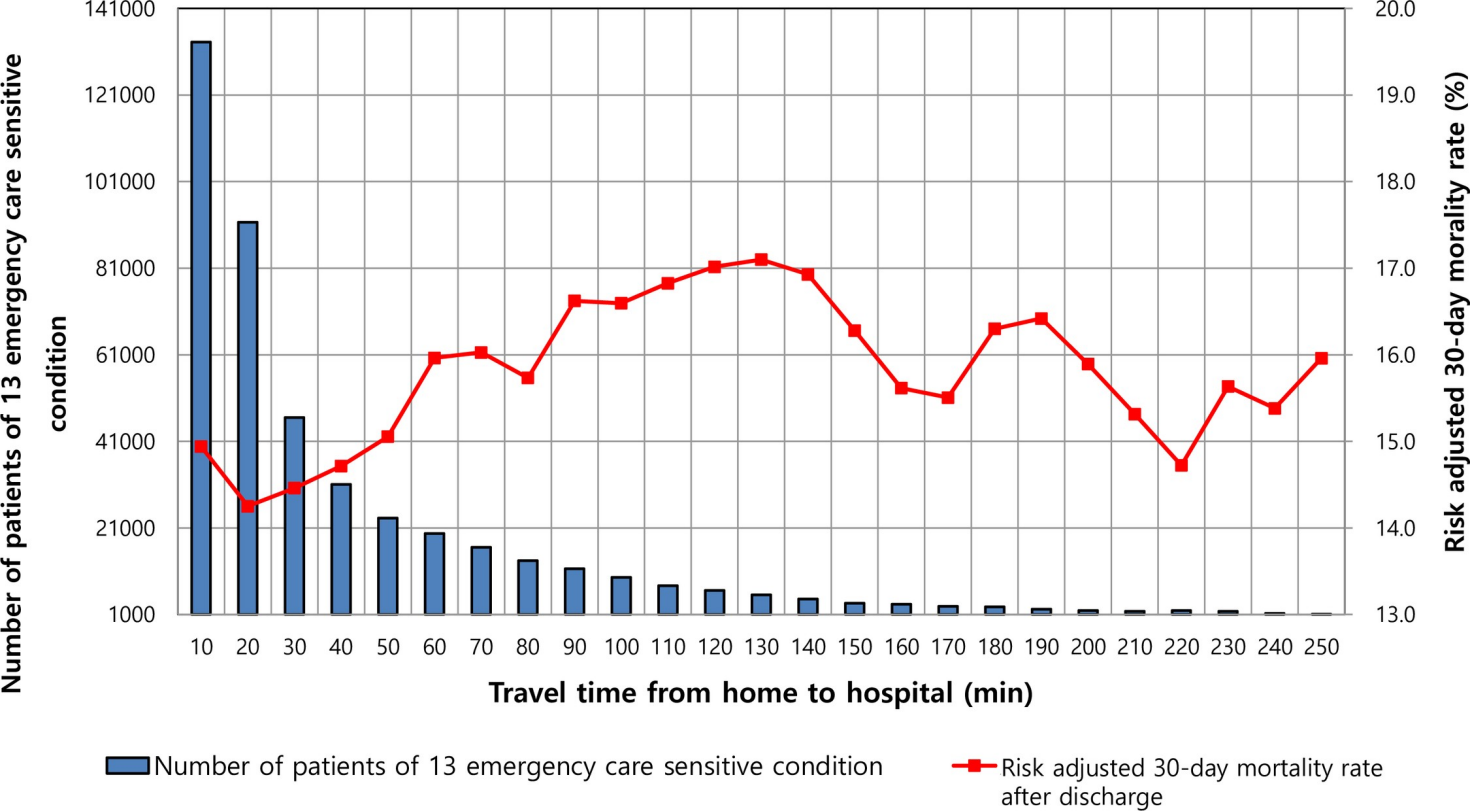
The number of patients and risk adjusted mortality rate according to access time to hospital in the thirteen emergency care sensitive conditions.

Overall, 61% of the patients resided within 30 min from the hospital; 78%, within 60 min; and 92%, within 120 min. Fig 1 shows how the distribution of the number of patients changes with increasing travel time to hospital.

Among the 13 ECSCs, 5 ECSCs had a significant threshold where the mortality changed. These were intracranial injury, AMI, other acute ischemic heart disease, fracture of the femur, and sepsis. The remaining 8 ECSCs did not show a significant change point of travel time to hospital. Table 3 shows the OHAT of the 5 ECSCs in confidence intervals (outcome variability) and confidence levels (CL). The OHAT of the 5 ECSCs ranged from 31 min to 80 min, with the fastest for AMI, and the slowest for intracranial injury. With respect to intracranial injury, the probability of mortality significantly changed at 71–80 min of travel time to hospital (99% CL). For AMI and other acute ischemic heart disease, the probability of mortality changed within 31–40 min (97% CL) and 70–80 min (97% CL) of travel time to hospital, respectively. The critical change points for fracture of the femur and sepsis were observed at 41–50 min and 61–70 min, respectively,

**Table 3:** Change points of adjusted 30-day mortality per ECSC.

ECSC	Significant change point	
	CP time (CI), min	CL
Intracranial injury	71–80	99%
Acute myocardial infarction	31–40	97%
Other acute ischemic heart disease	70–80	97%
Fracture of the femur	41–50	98%
Sepsis	61–70	100%

* CP time represents change point as outcome variable (confidence interval) with confidence level, as estimated with the CPA tool.

CP, change point; CI, confidence interval; CL, confidence level

**Fig 2:**
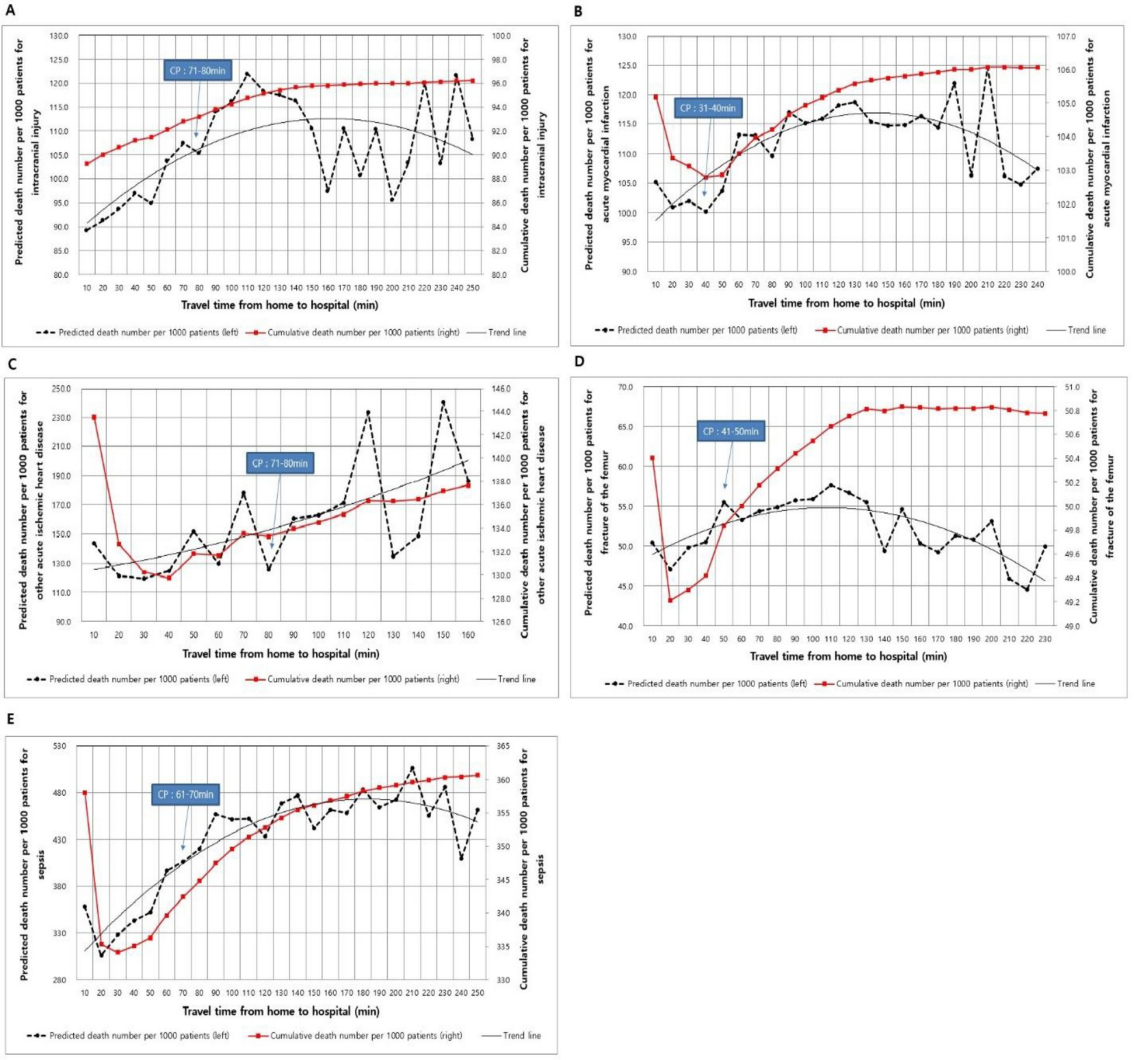
The risk adjusted mortality rate and access time to hospital in the five emergency care sensitive conditions. CP, change point.

Overall, there was a positive relationship between an increase in risk-adjusted mortality and increase in travel time to hospital (Fig 2). The risk-adjusted mortality rate initially decreased during the first 10 min and then increased until 150 min, with fluctuations. Thereafter, it slightly declined, except for intracranial injury and other acute ischemic heart disease. The U-shape mortality rate observed in the early phase is probably because death on hospital arrival was not excluded in from the number of deaths. This U-shaped mortality rate per 10 min in the early period was not observed in intracranial injury and no latter part slight decrease in other acute ischemic heart disease.

Across all 5 ECSCs, the relative mortality risk was 1.04–7.21 times higher for those living outside the OHAT than those living inside the OHAT (OR: 1.04–7.21, 95% CI: 1.03–26.34, Table 4). The difference in mortality risk was the largest among patients with other acute ischemic heart disease (OR: 7.21, 95% CI: 1.97–26.34). In these patients, those living outside the OHAT had 7.21 times higher mortality risk than those living inside the OHAT. Meanwhile, the difference in mortality was the smallest among patients with intracranial injury (OR: 1.04, 95% CI: 1.03–1.03). In these patients, those residing outside the OHAT had a 1.04 times higher mortality risk than those residing inside the OHAT.

**Table 4:** Odds ratio for the adjusted 30-day mortality by travel time on multiple logistic regression analysis*.

ECSC	Travel time	OR (95% CI)	p-value
Intracranial injury	71–80 min (ref: ≤70 min)	1.04 (1.03–1.05)	<0.0001
Acute myocardial infarction	31–40 min (ref: ≤30 min)	1.15 (1.12–1.18)	0.0093
Other acute ischemic heart disease	71–80 min (ref ≤70 min)	7.21 (1.97–26.34)	0.0028
Fracture of the femur	41–50 min (ref ≤40 min)	1.05 (1.03–1.07)	<0.0001
Sepsis	61–70 min (ref ≤60 min)	1.25 (1.06–1.47)	0.0067

*Adjusted for age, socioeconomic status, Carlson comorbidity index, ISS (only intracranial injury, fracture of the femur), urbanization, and hospital volume

OR, odds ratio; CI, confidence interval; p-value, probability value; min, minutes

## Discussion

Evidence of travel time benchmarks in view of the association between travel time and the outcomes of emergency care is limited. Our results showed that the OHAT for emergency care should be within the 31–80 min range, particularly for five ECSC (i.e., intracranial injury, AMI, other acute ischemic heart disease, fracture of the femur, and sepsis). Even after adjustment, there was a 1.04–7.21-fold difference in mortality between patients residing inside and outside the OHAT. To the best of our knowledge, this is the first study to establish the optimal access time to emergency medical care based on the travel time-outcome relationship in South Korea.

The estimated change points of mortality were consistent with those of previous studies for AMI, while there were some differences for the other ECSC. Among Swiss AMI patients who were aged over 65 years, those who lived more than 29 min to the university hospital had a relatively higher mortality than did patients who lived within 29 min to the hospital [9]. In addition, stroke patients who were aged at least 65 years and lived more than 19 min to the central hospital were more likely to die than those who lived within 19 min. In Canada, more than 30 min of travel time to emergency care resulted in worse mortality than less than 30 min of travel time among patients with severe injuries [11]. Although travel time to emergency care may be affected by road and traffic conditions, efforts should be made to ensure travel time to hospital within a minimum of 30 min and a maximum of 80 min to optimize the outcomes of emergency treatment. It is essential to strategically allocate public resources to improve the mortality of patients living far from emergency medical centers.

We also identified five ECSCs for which patients living outside of the OHAT had a 1.04–7.21 times higher risk of mortality than those living within the OHAT. These were ischemic heart disease, AMI, brain injury, sepsis, and fracture of the femur. Many studies have shown the reverse associations between the survival probability of time-sensitive cardiovascular disease and geographical distance to emergency medical centers [8–10, 12, 27]. The adverse mortality effects of a long travel distance to hospital were also observed in cases of sepsis and injuries (e.g., traumatic brain injury) [11, 28, 29]. In addition, our risk-adjusted analysis also suggested that distance decay affects the survival probability of these five ECSCs. This highlights the need for establishing and validating the OHAT for emergency care. However, we failed to observe the effects on the remaining 8 ECSCs. There is a lack of strong evidence supporting high mortality in time-sensitive diseases due to low geographic access. The association between increased distance to hospital and mortality in diseases requiring emergency care [8, 30, 31] needs to be further investigated.

Since 2017, the South Korean government has identified areas with more than 30 min driving time to local emergency medical centers or 60 min to regional emergency medical centers as vulnerable areas under the Public Health and Medical Services Act. However, prior research to examine the evidence of driving time thresholds for the classification of vulnerable areas in South Korea is lacking. To resolve the disparities in geographical accessibility to emergency care, the optimal distance between emergency medical centers in the community and the obstacles to achieving this needs to be discussed and verified.

Changes to the delivery of emergency services sometimes hinder the improvement of the emergency care system. However, the effects of emergency service reconfiguration on the relationship between travel time to hospitals and mortality of ECSC are still debated [8, 10, 30–32]. When based on the volume-outcome relationship, reconfiguration of emergency care should involve focusing resources on hospitals with specialty for severe acute conditions to provide high-quality services and improve outcomes. However, the results for such strategy are conflicting. Some studies have shown that reconfiguration has negative effects on ECSC mortality by reducing geographic accessibility, overcrowding emergency rooms, and increasing patient cost [8, 10, 32]. In contrast, other studies have presented evidence that reconfiguration has no significant effects on mortality as it increases investment in emergency services and preparation of remaining nearby centers [30, 31]. The problem of the geographic accessibility gap needs to be further investigated to identify OHAT based on robust evidence that can explain the opportunity to enhance the performance of the system and to predict the consequences of the changes. As presented in the current study, the South Korean government needs to plan and position emergency medical centers within 80 min not by geographic time but by optimal time for outcomes. Travel time within 80 min can be used as a standard minimum requirement of response to resolve the disparities in geographical accessibility to emergency care. Therefore, it is necessary to deploy emergency medical centers within 80 mins of travel time especially in vulnerable areas. However, in regions with abundant emergency care resources, other solutions can be considered, such as reorganization based on a volume-outcome relationship. This study has some notable limitations. First, there may be differences between actual transportation information (e.g., time, means, etc.) and the proxy transportation information (e.g. address-based travel time, assumption of motor vehicle use, etc.). Second, the outcome measure was in-hospital mortality, which could not explain out-of-hospital deaths and longer period mortality. This may have resulted in an underestimation of the impact of distance decay on ECSC mortality. Third, we used the database comorbidity scale, instead of information on clinical severity, due to data limitations. Therefore, the actual severity of the acute conditions may have been underestimated. Fourth, this was a cross-sectional study, and thus, could not determine the causal associations between travel time and mortality of ECSC.

## Conclusion

The OHAT for emergency care with no significant increase in mortality is in the 31–80 min range. Patients living outside of the OHAT had a 1.04–7.21 times higher mortality risk than those living inside the OHAT, even after adjustment. Our findings indicate that emergency medical centers should be positioned within a minimum of 30 min and a maximum of 80 min not by geographic time but by optimal time for outcomes.
